# Growing Healthy Hearts: a single-arm feasibility study of a digitally delivered gardening, cooking, and nutrition intervention for adults with risk factors for cardiovascular disease

**DOI:** 10.1186/s40814-023-01380-5

**Published:** 2023-08-31

**Authors:** Susan Veldheer, Maxfield Whitehead-Zimmers, Candace Bordner, Olivia Weinstein, Hena Choi, Kira Spreenberg-Bronsoms, Jason Davis, David E. Conroy, Kathryn H. Schmitz, Christopher Sciamanna

**Affiliations:** 1 Department of Family and Community Medicine, Penn State College of Medicine, Hershey, PA 17033 USA; 2 Department of Public Health Sciences, Penn State College of Medicine, Hershey, PA USA; 3 Boston Medical Center, Boston, MA USA; 4 Department of Nutritional Sciences, Penn State University, State College, PA USA; 5 Department of Early, Middle, and Exceptional Education, Millersville University, Millersville, PA USA; 6 Department of Kinesiology, Penn State University, State College, PA USA; 7 University of Pittsburgh School of Medicine, Pittsburgh, PA USA; 8 Department of Medicine, Penn State College of Medicine, Hershey, PA USA

**Keywords:** Cardiovascular disease risk, Gardening, Multiple behavior change, Dietary intake, Physical activity, Feasibility study

## Abstract

**Background:**

Food gardening may positively influence cardiovascular disease (CVD) risk-related behaviors. However, the vast majority of existing gardening interventions have used an in-person delivery model which has limitations for scalability. It is not known whether a digitally delivered gardening intervention would be feasible or acceptable to participants. The purpose of this pilot study was to assess the feasibility of a digitally delivered gardening intervention in three domains: participant acceptability, demand, and practicality.

**Methods:**

A single-arm, pre-post-study design was used. Participants (*n* = 30) were aged 20 + with no plans to garden in the coming season and had at least 1 CVD risk factor. The intervention included ten 1-h video-conferencing sessions, written materials, and access to a study website. Content focused on gardening skills, cooking skills, and the Dietary Approaches to Stop Hypertension (DASH) diet. Feasibility outcomes included acceptability (post-program ratings), demand (session attendance rate), and practicality (ability to start a garden and grow F&V). The study was considered feasible if the following criteria were met: ≥ 70% rated the intervention as good or excellent, overall session attendance rate was ≥ 70%, and > 70% were able to start a garden and grow F&V. We also assessed pre-post**-**program changes in behavioral mediators (gardening confidence, gardening enjoyment, cooking confidence, and nutrition knowledge). Descriptive statistics were calculated. Pre-post differences were evaluated with means and 95% confidence intervals (95% CI). Effect sizes were calculated (Cohen’s d).

**Results:**

All feasibility criteria were met. A total of 93.3% of participants rated the intervention as good or excellent, 96% started a garden and grew F&V, and the overall session attendance rate was 81%. The largest mean pre-post changes were in gardening confidence (pre 7.1 [95% *CI*: 6.4, 7.9], post 9.0 [95% *CI*: 8.6, 9.5], Cohen’s *d* = 1.15), gardening enjoyment (pre: 6.3 [95% *CI*: 5.9, 6.7], post: 7.5 [95% *CI*: 7.1, 7.9], Cohen’s *d* = 1.69), and cooking self-efficacy (pre: 4.7 [95% *CI*: 4.3, 5.1], post: 7.7 [95% *CI*: 7.3, 8.0], Cohen’s *d* = 3.0).

**Conclusion:**

A digitally delivered gardening intervention was feasible, acceptable to participants, and they had meaningful changes in behavioral mediators. The next step is to evaluate the impact of the intervention in a future randomized controlled trial.

## Key messages regarding feasibility

- The vast majority of gardening-related interventions have been delivered using in-person delivery modalities. It is unknown if a digitally delivered intervention would be acceptable and feasible for adults with risk factors for cardiovascular disease.
- This study found that a digitally delivered gardening intervention was feasible in three domains including acceptability measured by post-program participant evaluation, demand for the intervention as measured by overall attendance rate, and practicality as measured by the ability of participants to start a garden and grow fruits and vegetables.
- The findings from this study support the need for further evaluation of the Growing Healthy Hearts gardening intervention using a randomized controlled trial design.

## Introduction

Cardiovascular disease (CVD) is the leading cause of premature death in the USA [1]. Healthy lifestyle behaviors such as fruit and vegetable (F&V) intake and adequate physical activity can reduce the risk of premature CVD-related mortality by 12–23% [2, 3]. However, the number of Americans who report meeting the minimum daily requirements of 5 F&V servings/day is low at just 12% [4]. Similarly, just 23% meet recommended levels of aerobic physical activity (PA) and muscle-strengthening activities [5]. These data suggest that new and innovative interventions are needed to influence the multiple health behaviors that impact CVD risk.

One possible intervention that could contribute to better CVD health status is food gardening (herein referred to as gardening). Observational studies have found that gardeners are 2–3 times more likely than non-gardeners to consume at least 5 servings of F&V/day [6, 7]. In addition, gardening includes several moderate to vigorous intensity physical activities from walking (metabolic equivalent task [MET] 3.0), to raking (*MET* 3.8), to digging (*MET* 5.0), and pushing a wheel barrow (*MET* 5.5) [8]. Taken together, these data provide support for the idea that teaching gardening may be a reasonable intervention strategy to help adults with CVD risk factors meet recommended F&V intake and physical activity goals.

Currently, the vast majority of research on the benefits of gardening is observational [9, 10]. In addition, despite the potential application of gardening for CVD risk reduction, the only published randomized controlled trials (RCTs) that prospectively teach gardening skills have been conducted in two specialized populations: school-aged children [11, 12] and adult cancer survivors [13, 14]. Furthermore, these studies have all used in-person delivery modes which can present participation barriers such as the need for transportation and child care [15, 16]. Digitally delivered interventions can address participation barriers and increase the potential for large-scale dissemination. To our knowledge, there are no digitally delivered gardening interventions specifically designed to address health outcomes or CVD risk in adults.

We previously developed an in-person, multiple behavior change gardening intervention designed to impact dietary intake of F&V and PA among adults with risk factors for CVD [17]. Recognizing the limitations of the in-person intervention delivery model, and with the goal of increasing future scalability, the intervention was adapted to be digitally delivered via video-conferencing (Zoom) and web-based materials. Prior to moving to a randomized trial, pilot testing is necessary to assess whether a digitally delivered gardening intervention would be feasible or acceptable to adults with CVD risk factors. Therefore, we conducted a single-arm pilot study to assess feasibility in three domains as outlined by Bowen et al. [18]: acceptability, demand, and practicality. In addition, we assessed preliminary outcomes for pre-post changes in F&V intake, PA, and other behavioral factors.

## Methods

### Study design, participants, and intervention

**Fig. 1:**
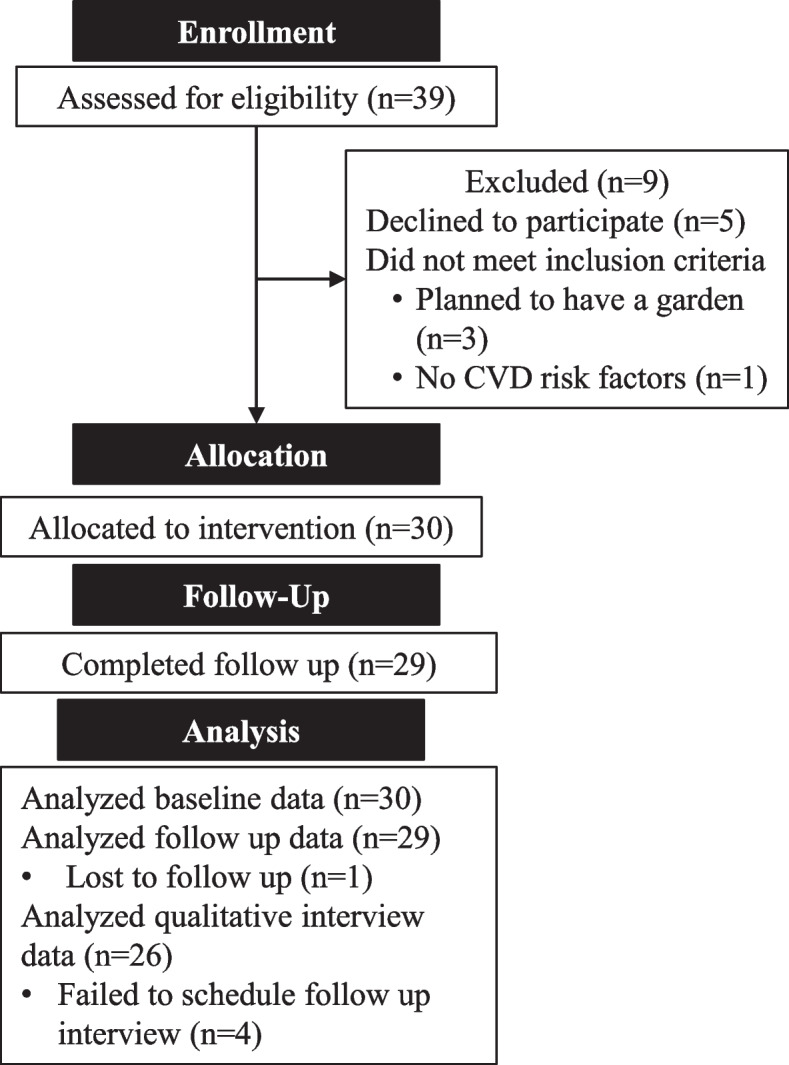
CONSORT diagram of participant flow through the study

This was a prospective, 21-week, single-arm pre-post-study design with outcomes assessed at baseline, midpoint, and follow-up. Adults aged 20 + years who attended a primary care clinic in Hershey, PA, USA, in the past year were invited to the study via email. Interested participants were screened over the phone between March 30, 2021, date and April 21, 2021. Eligible participants were those who: (1) had no plans to start or tend a garden in the coming season and (2) had a self-reported history of at least one of the following cardiovascular risk factors: body mass index (BMI) ≥ 25 kg/m^2^, hyperlipidemia, hypertension, diabetes, stroke, current or past tobacco use, and family history of heart attack before age 50. Additional criteria included access to reliable Internet and access to garden space (in ground, raised beds, or pots; at home or a community garden). When screening for space, the study team member discussed all possible locations that the participant had access to, and they were included if this was any 1, or a combination of, containers, raised beds, in the ground, or a plot at a community garden. Once eligibility was established, participants provided written consent for study participation using an institutional review board (IRB)-approved remote consent process that used a combination of REDCap and videoconferencing. Details of participant flow through the study are presented in Fig. 1.

In terms of sample size, one reason to conduct a pilot study is for the study team to test the trial procedures. In the present study, this included digital intervention delivery in addition to remote consenting and data collection [19]. Because of the seasonal nature of gardening, we used a cohort group structure where all participants enrolled, completed data collection, and engaged in the intervention at the same time points. Therefore, the sample size of 30 was chosen for 3 reasons of practicality [19, 20]: (1) this group size was the maximum considered operationally feasible for simultaneous participant data collection at each time point, (2) it was reasonable for managing participants during the live video-conferencing sessions, and (3) this is an acceptable number needed to demonstrate participant acceptability, demand, and feasibility [19, 21–23].

The majority of the intervention sessions focused on gardening skills. However, the cooking and nutrition education assisted participants with a full lifecycle overview of gardening as a healthy lifestyle activity encompassing growing, preparing, cooking, and eating fresh F&V (i.e., garden to plate). The theoretical framework guiding the intervention was social cognitive theory (SCT) and self-determination theory (SDT) [24, 25]. Social cognitive theory suggests that knowledge and self-efficacy are important psychosocial mediators of health behavior change [25, 26]. In this case, nutrition knowledge and cooking self-efficacy were targeted due to their associations with better overall diet quality [27]. In addition, SDT suggests that when people are able to maintain behaviors for the long term, it is due to internal motivation via psychosocial need satisfaction. Internal motivation is increased when people have autonomy and feel competent in performing behaviors. This in turn contributes to feelings of enjoyment and satisfaction [24, 28, 29]. Therefore, the mediators targeted were gardening enjoyment and gardening confidence.

The intervention included ten 1-h videoconferencing sessions via Zoom (7 gardening focused and 3 cooking focused). Although the intervention was delivered digitally, participants engaged in the gardening and cooking activities in their own gardens and kitchens. All gardening sessions included 10–15 min with a registered dietitian (SV) presenting on topics such as using the Dietary Approaches to Stop Hypertension (DASH) diet [30–32], MyPlate, portion sizes, grocery shopping, meal planning, and label reading. Prior to starting the sessions, participants were mailed 2 hard-copy booklets (1 nutrition focused and 1 gardening focused). Because soil nutrient composition and Ph are important for gardening success, all participants also received a prepaid (US $9.00) soil test kit from the Penn State Extension. Instructions for taking the sample, sending it to the lab for analysis, and interpreting the results were provided as part of the gardening instruction. Sessions were conducted in three phases. Phase 1 included 5 weekly sessions with Penn State Extension certified Master Gardeners. Training for a Master Gardener certification in Pennsylvania includes 40 h of classroom instruction, passing a written exam, and completing 50 h of garden-focused service. Topics for the sessions included the following: gardening skills to assist the participants with identifying a garden site (in ground, in containers, raised beds, or in a community garden), preparing the site for planting, tool safety, general planting (spring and summer gardens), garden maintenance, and pest control. Phase II, held biweekly, included three cook-along sessions where participants were provided a DASH diet-appropriate, garden produce-focused recipe in advance and were invited to cook along with a culinary medicine dietitian (OW). These sessions focused on incorporating garden produce and herbs into basic meals such as sandwiches, salad or grain bowls, and stir fries, or pasta dishes. The last two sessions during Phase III were held monthly, and Master Gardeners focused on starting a fall garden, storing the harvest, and garden cleanup. Each session began and ended with a question and answer period where participants were encouraged to share their successes and challenges with the group. During the study, participants received access to a study website that included additional materials on topics covered during the sessions (i.e., gardening instructional videos, DASH nutrition information, and healthy recipes).

Based on the TIDieR checklist and the behavior change wheel [33, 34], details about the intervention are presented in Table 1 including the intervention components, intervention functions, content, content delivery modes, time spent, and who presented each session.

**Table 1:** Growing Healthy Hearts digitally delivered gardening intervention description for each component

Intervention function^a^, content, time spent, session presenter	Delivery mode^b^				
	VC	BK	WS	GC	GD
**Gardening component** Online materials, written booklet, and 7–20- to 30-min presentations with Master Gardeners^c^					
**Education**: Information about gardening activities and maintenance	x	x	x		x
**Training**: Instruction on how to perform gardening tasks (site selection, garden installation, planting, weeding, pest control, watering, harvesting, and cleanup)	x	x	x		x
**Enablement**: Provide money for new supplies (US $100)^d^				x	
**Environmental restructuring**: Garden installation, problem solve, and provide social support (group discussion and sharing successes)^e^	x	x			x
**Cooking component** Online recipes, written booklet, and 3 1-h cook-along sessions with a culinary dietitian^c^					
**Education**: Information about the health benefits of a healthy cooking practices	x				x
**Training**: Cook-along sessions with pre-set recipes using garden produce	x				
**Enablement**: Provide garden-focused recipes		x	x		
**Environmental restructuring**: Problem-solve and provide social support (group discussion and sharing successes)^e^	x				x
**Nutrition education component** Online and written materials, 7–10- to 15-min presentations with a registered dietitian^c^					
**Education**: Information about the benefits of the DASH^b^ diet for cardiovascular health	x	x	x		
**Training**: Using DASH with MyPlate, food labels, meal planning, and shopping for F&V on a budget	x				
**Environmental restructuring**: Problem-solve and provide social support (group discussion and sharing successes)^e^	x				x

^a^Intervention functions categorized based on the behavior change wheel from Michie et al.

^b^Abbreviations and descriptions: *VC* videoconferencing with or without power point slides, *BK* booklet, *WS* website with videos and written materials, *GC* gift card, *GD* group discussion via question and answer time with experts, *DASH* dietary approaches to stop hypertension, *F&V* fruits and vegetables

^c^Behavior change wheel intervention function of persuasion using a credible source

^d^GC was unrestricted and could be used for cooking, gardening, or any other expenses

^e^Group discussion and sharing successes were allocated 10–15 min during all sessions. Topics were participant driven and open to any content area

Participants were compensated US $160 for their study participation which included US $20 for completing surveys at each time point and an additional US $100 which could be used to purchase gardening supplies. This study was approved by the Penn State University IRB (protocol no. 17020).

### Measures

Participants completed Research Electronic Data Capture (REDCap) surveys at each of the three timepoints (baseline, midpoint 10 weeks, and follow-up 21 weeks) over 21 weeks in 2021 (April-September). The post-program acceptability rating was assessed with the question “Overall, how would you rate the gardening program that you participated in?” Acceptability was defined as ≥ 70% of participants rating the intervention as good/excellent (vs. satisfactory/poor). Demand was based on overall attendance rate with a goal of 70% participation. This conservative approach for attendance takes into consideration all possible participants (*n* = 30) attending all possible sessions (*n* = 10) and is calculated as the total attendance for all sessions (actual)/the maximum possible for all sessions (300). Individual session attendance was also assessed as the number and percent who attended at least 7 sessions (i.e., received 70% of the intervention). Practicality was assessed post-program, and participants were asked to rate, on a scale from 1 (disagree) to 10 (agree), to what degree they were able to “start a garden” and “grow fruits and vegetables.” The intervention would be considered practical if at least 70% of participants provided a high rating of ≥ 8 for both questions. An additional practicality measure using the same scale was used to assess 10 key gardening program activities with a goal of least 70% of participants providing a high rating (≥ 8) for 7 of 10 activities including the following: choosing a site; completing a soil test; starting a spring, summer, and fall garden; weekly garden tending; controlling pests; weeding; harvesting; and incorporating garden produce into meals. Areas for programmatic improvement were solicited in a post-program recorded video-conferencing exit interview where participants were asked, “If we did this program again, what do you think should be done differently and why?”.

PA was assessed using the International Physical Activity Questionnaire (IPAQ) long form [35]. Standard analytical guidelines were used to estimate PA in MET minutes (minutes of activity × the assigned MET value for each reported activity) [35]. Dietary intake was measured with the 26-item National Health and Nutrition Examination Survey (NHANES) dietary screener questionnaire (DSQ) [36]. The NHANES DSQ analytical guidelines were used to generate estimates for daily servings of F&V without French fries (cup equivalents), fiber (grams), dairy (cups), total and added sugars (teaspoons), whole grains (grams), and calcium (mg) [36]. Due to a technical error, this measure was only collected at baseline and follow-up. Cooking confidence was measured with the Cooking and Food Provisioning Action Scale (CAFPAS) functional sub-scale, a 13-item questionnaire which assesses an individual’s self-perceived cooking confidence using a 7-point scale (1 = disagree and 7 = agree) [37]. Nutrition knowledge was assessed with 25 questions from two domains of the General Nutrition Knowledge Questionnaire (dietary recommendations and food groups) [38]. Questions in these domains were modified to align with DASH diet and MyPlate recommendations. Each correct answer was summed for a total score of 25 with higher scores indicating higher nutrition knowledge. Gardening enjoyment was assessed using a modified CAFPAS attitudes sub-scale (10 items) where references to cooking were replaced with gardening [37]. Questions asked participants to indicate to what degree they agree (1 = disagree to 7 = agree) with statements related to personal fulfillment and enjoyment such as “I find gardening a very fulfilling activity” or “Compared to other activities, gardening brings me little enjoyment.” Five items for gardening confidence (10-point scale, 1 = not at all confident, 10 = very confident) were used to assess confidence in the following: starting a garden, watering, growing produce from seed, growing produce from established plants, and harvesting. Questions were summed and reported as a mean score. Mental health was assessed with the PROMIS depression and anxiety scales (presented as *t*-scores) [39, 40] and the Perceived Stress Scale [41]. Participants were also asked (yes/no), “Would you recommend this program to family or friends” and “would you consider growing a garden next season?”.

### Data analysis

Summary statistics were calculated for quantitative data. Changes in behavioral outcomes from baseline to each timepoint were assessed with means and 95% confidence intervals. Statistical analysis was conducted using SAS statistical software version 9.4 (SAS Institute, Cary, NC, USA). For qualitative interviews, a codebook was developed, and improvements for a future intervention were coded by 2 study team members (Cohen’s kappa = 0.83) using NVivo version 12 (QSR International, Melbourne, Australia). Codes were grouped into themes and organized by intervention component (gardening, cooking, nutrition education).

## Results

Baseline participant sociodemographic and health characteristics (*n* = 30) are presented in Table 2. The sample was majority white and female, and the highest proportion of CVD risk factors was high BMI, hypercholesterolemia, and hypertension.

**Table 2:** Growing Healthy Hearts participant demographic and health-related characteristics (*n* = 30)

Characteristic	Mean or % (*n*)
**Age, mean [SD]**^**a**^	49.9 [15.2] (30)
**% female**	83.3 (25)
**% male**	16.7 (5)
**% Hispanic or Latino**	3.3 (1)
**% Not Hispanic or Latino**	96.7 (29)
**Race**	
% white	80.0 (24)
% African American	10.0 (3)
% Asian	10.0 (3)
**% Education level, less than bachelor’s degree**	30.0 (9)
**Employment status**	
Working full- or part-time	63.3 (19)
Retired	23.3 (7)
Any other	10.4 (4)
**% married or living as married**	63.3 (19)
**% not married or living as married**	36.7 (11)
**% residence is a house**	86.7 (26)
**% residence, an apartment or other**	13.3 (4)
**% yes, any food insecurity in past 12 months**	10.0 (3)
**% no food insecurity in past 12 months**	90.0 (27)
**% who have used any social services, past 5 years**^**a**^	23.3 (7)
**% used no social services, past 5 years**	76.7 (23)
**Cardiovascular disease risk factors**^**b**^	
% yes, ever told diabetes	23.3 (7)
% never told diabetes	76.7 (23)
% yes, ever told high blood pressure	53.3 (16)
% never told high blood pressure	46.7 (14)
% yes, ever told high cholesterol	63.3 (19)
% never told high cholesterol	36.7 (11)
% yes, ever told heart disease	3.3 (1)
% never told heart disease	96.7 (29)
% yes, family history of early heart attack	20.0 (6)
% no history of early heart attack	80.0 (24)
% yes, ever told stroke	6.7 (2)
% never told stroke	93.3 (28)
% yes, overweight or obese (body mass index ≥ 25 kg/m^2^)	76.7 (23)
% no overweight or obese (body mass index < 25 kg/m^2^)	23.3 (7)
% yes, any current or past tobacco use	33.3 (10)
% no current or past tobacco use	66.7 (20)

^a^Abbreviations: *SD* standard deviation

^b^Responses were not mutually exclusive, and participants could self-report all that apply

Follow-up data were collected on 29/30 participants (96.6% follow-up rate). Feasibility criteria for each domain are presented in Table 3. Predetermined criteria were met or exceeded for each domain (acceptability, demand, and practicality). In terms of overall program acceptability and future intentions, 100% of participants (*n* = 29) reported both that they would recommend the program to family or friends, and that they would consider growing a garden next season. In terms of individual session attendance, 26/30 participants (86.6%) attended at least 7 sessions. For individual gardening activities, the three items that had the lowest ratings were starting a spring garden, a fall garden, and completing the soil test.

**Table 3:** Post-program feasibility outcomes (*n* = 29)

Feasibility outcome and program goal	Goal achieved (yes/no), % (*n*)
**Acceptability**: Post-program rating of good/excellent (v. satisfactory/poor) from at least 70% of participants	Yes, achieved for 93% of participants (27)
**Demand**: Overall session attendance rate of 70%	Yes, achieved 81% overall attendance rate^a^
**Practicality**: At least 70% of participants were able to start a garden and grow fruits and vegetables	Yes, achieved for 96% of participants (28)
**Individual practical gardening activities**: At least 70% of participants completed 7 of 10 activities	Yes, achieved for 86% (25)
Choosing a garden site	Yes, 100% (29)
Completing the soil test	No, 59% (17)
Starting a cool season (spring) garden	No, 55% (16)
Starting a warm season (summer) garden	Yes, 93% (27)
Starting a fall garden	No, 51% (15)
Tending the garden weekly	Yes, 86% (25)
Controlling garden pests	Yes, 76% (22)
Weeding	Yes, 90% (26)
Harvesting from the garden	Yes, 93% (27)
Incorporating garden produce into meals	Yes, 90% (26)

^a^Overall session attendance rate based on 30 participants attending 10 sessions (244/300 = 81%)

Changes in behavioral mediators and health outcomes from baseline to each timepoint are presented in Table 4. The largest mean pre-post changes were in gardening confidence, gardening enjoyment, and cooking self-efficacy.

**Table 4:** Study outcomes and effect sizes for differences at baseline to midpoint and baseline to follow-up

	**Baseline**	**Midpoint (week 10)**	**Cohen’s d**^**a**^	**Follow-up (week 21)**	**Cohen’s d**^**b**^
**Gardening confidence, mean (95% CI)**^**c**^	7.1 (6.4, 7.9)	8.6 (8.2, 9.1)	0.89	9.0 (8.6, 9.5)	1.15
**Gardening enjoyment, mean (95% CI)**	6.3 (5.9, 6.7)	7.2 (6.9, 7.6)	0.90	7.5 (7.1, 7.9)	1.69
**Cooking self-efficacy, mean (95% CI)**	4.7 (4.3, 5.1)	6.8 (6.5, 7.2)	2.10	7.7 (7.3, 8.0)	3.00
**Nutrition knowledge score, mean (95% CI)**	23.3 (22.8, 23.8)	23.6 (23.0, 24.2)	0.21	23.8 (23.3, 24.3)	0.37
**Dietary intake per day**					
Fruit and vegetable intake (cup equivalents), mean (95% CI)^d^	2.4 (2.2, 2.7)	-	-	2.8 (2.6, 3.1)	0.73
Fiber (g), mean (95% CI)	15.5 (14.5, 16.5)	-	-	16.8 (15.7, 17.9)	0.45
Dairy intake (cups), mean (95% CI)	1.5 (1.4, 1.7)	-	-	1.3 (1.2, 1.4)	0.53
Total sugars (teaspoons), mean (95% CI)	15.2 (13.1, 17.4)	-	-	13.4 (12.1, 14.7)	0.38
Added sugars (teaspoons), mean (95% CI)	5.7 (3.9, 7.4)	-	-	4.8 (4.2, 5.4)	0.26
Whole grains (g), mean (95% CI)	0.7 (0.6, 0.8)	-	-	0.7 (0.6, 0.8)	0.00
Calcium (mg), mean (95% CI)	931.3 (862.5, 1000.1)	-	-	890.4 (844.7, 936.0)	0.26
**Physical activity in MET minutes, mean (95% CI)**	3082 (2304.0, 3861.3)	4229 (2971.5, 5485.8)	0.41	3405 (2375.7, 4435.6)	0.13
**Mental health outcomes**					
Perceived stress, mean (95% CI)	12.5 (9.6, 15.5)	13.7 (10.9, 16.5)	0.16	11.8 (9.2, 14.4)	0.09
PROMIS anxiety, mean (95% CI)^e^	51.4 (47.5, 55.2)	49.5 (46.0, 53.1)	0.19	49.0(45.4, 52.6)	0.24
PROMIS depression, mean (95% CI)^e^	50.5 (46.9, 54.1)	46.9 (43.5, 50.3)	0.38	47.4 (43.7, 51.2)	0.32

^a^Represents effect size from baseline to midpoint

^b^Represents effect size from baseline to follow-up (*n* = 29)

^c^Abbreviations: 95% *CI* 95% confidence interval, *MET* metabolic equivalent tasks

^d^Estimated fruit and vegetable intake per day without French fries

^e^Reported as a *t*-score, calculated based on PROMIS analytical guidelines

Results of the exit interviews (*n* = 26), including participant recommendations for each intervention component and illustrative quotes, are presented in Table 5. Participants recommended Master Gardener presentations include in-garden skills demonstrations and a slower pace for the cooking demonstrations. They also suggested more group interaction (either online or in person); more advanced skills training for specific gardening, cooking, and nutrition topics; and guidance on modifying nutrition and cooking information based on personal or cultural preferences.

**Table 5:** Participant future program recommendations with illustrative quotes from post-program exit interviews

**Gardening component**
**Consider hybrid or in-person sessions**: “I would prefer hybrid or all in person”; “Get together and actually see a garden live” **Add educational content**: “More help with the tools”; “More time spent on pests and pest control” **Add skills training**: “A garden planner to plot out the garden” **Include in-garden demonstrations**: “Have master gardeners in the garden instead of in their living room” **Provide more opportunities for group interaction**: “Meet the people that you're in a community with to get advice”
**Cooking component**
**Provide recipe swaps and substitutions**: “Have some substitutions for those with allergies…Or if you don't like this, you can use this” **Conduct cook-along at a slower pace**: “I didn't cook along. I didn't move fast enough to do that” **Add more advanced cooking skills**: “Some sessions were on the elementary side for someone [experienced with cooking]”
**Nutrition education component**
**Offer more advanced nutrition topics**: “I don't know if I really learned anything new about nutrition. It reinforced the things that I should be doing, like less sodium” **Incorporate more cultural diversity**: “The nutrition presentations were generic. It didn't get much diversity of people's backgrounds”

## Discussion

This single-arm feasibity study of a digitally delivered, integrated gardening, cooking, and nutrition education program found that the intervention was feasible in three domains: acceptability, demand, and practicality. The digitally delivered intervention had an acceptability rating that was similar to the existing in-person version (93% rated highly) [17]. In addition, we had a high attendance rate over the 21-week study period, and participants were able to complete a range of garden to plate activities including site selection, planting, harvesting, and eating fresh produce from their gardens. Results of this study suggest that a digitally delivered version of the intervention can be used in the future to increase the potential for scale and reach.

To the authors’ knowledge, this report is the first digitally delivered gardening intervention designed to improve health outcomes. With a focus specifically on CVD, we found evidence that a gardening intervention could be a tool to impact health through behavioral mediators including gardening enjoyment and confidence and cooking self-efficacy. There was also a moderate to high positive effect on F&V intake (Cohen’s *d* = 0.73) which is known to influence CVD risk [9, 42–44]. Epidemiologic studies have found an inverse, dose relationship between CVD mortality and F&V intake [2] with estimates suggesting a 4% reduction in CVD mortality for each additional serving of F&V consumed (up to 5 servings per day) [45]. Similarly, there is a linear dose relationship between time spent engaged in PA of any intensity and CVD risk reduction [3]. The effect on PA at the 21-week follow-up was nonsignificantly small (Cohen’s *d* = 0.13). However, given that gardening has been found to impact both diet and PA [6, 7, 13, 14, 46], further examination is warranted to assess whether it could impact these health outcomes in a larger sample of adults with one or more CVD risk factors.

The findings from this study are broadly consistent with the adult-focused gardening interventions in cancer survivors despite them being delivered using in-person modalities [13, 14]. For instance, Bail et al. randomized 82 breast cancer survivors with low baseline F&V intake (< 5 servings/day) and PA (< 150 min/week) into an in-person Master Gardener mentored (1:1) home garden-based intervention (*n* = 42) versus a wait-list control (*n* = 36) [14]. Compared to baseline, at follow-up, intervention participants had a 0.86 serving/day increase in vegetables (*p* < 0.001) and an increase in PA of 14 min/week (*p* = 0.34). Like the present study, Bail et al. also had a high acceptability rating (100%) with all participants indicating a willingness to garden in the next season. The authors concluded that gardening programs offer an integrated approach to improving health behaviors and that larger studies are needed to confirm these results.

There are many studies that have demonstrated the benefits of gardening on mental health [9, 10, 47]. In the present digitally delivered intervention, we found small to moderate effects on depression outcomes based on self-reported measures (Cohen’s *d* = 0.32). Mechanisms for why gardening may impact mental health are unclear, although there is an emerging body of literature which supports the idea that exposure to nature, or feelings of wonder and awe, may play a role in better mental and physical health [48, 49]. Although the evidence is mixed, it is possible there is a seasonal effect on mental health symptoms [50–52]. Since the baseline mental health measures were collected in late winter/early spring (March/April) and follow-up measures were collected in the late summer (August/September), parallel-group randomized controlled trials are needed to further elucidate this issue.

From the perspective of health equity and health disparities, the need for garden space could be viewed as a limitation. However, scholars and community organizers consider gardening to be both a means of personally procuring low-cost F&V (food justice) and a tool for promoting health equity and social justice [53–56]. This is in part because gardens are inherently flexible in terms of space (in ground, raised beds, containers, community gardens) and the produce that can be grown which supports individual autonomy and cultural diversity. In our program, Master Gardeners offered growing techniques for participant-chosen garden spaces while also offering strategies to accommodate different physical abilities (i.e., tall raised beds to eliminate the need to stoop or bend, ergonomic tools for better grip). Furthermore, participants chose what they wanted to grow, and the cooking sessions provided suggestions for swapping ingredients based on their preferences (although participants wanted more of this in the future).

This study has some limitations. First, it had a small sample size and no control group so conclusions about efficacy are not appropriate. A larger, adequately powered randomized controlled trial is necessary to fully assess the influence of gardening on CVD-related health behaviors and risk. Second, participants in the present study were selected based on a variety of CVD risk factors but not baseline dietary intake or physical activity levels. Thus, some participants may have already been consuming high levels of F&V or engaged in adequate PA, and this may have limited the amount of change that could be detected at follow-up. Future studies should include only participants who have low baseline levels of F&V intake and PA in addition to other CVD-risk factors. In terms of dietary outcomes, they were self-reported and were not measured at the midpoint due to a technical error. Objective measures of dietary intake such as plasma carotenoids, or more intensive device-based physical activity measures, would increase the rigor of future work. More frequent measurements of both dietary intake and PA would assist in providing a picture of how these outcomes may change over the course of the garden season. For our practically measures, participant responses were skewed to the high and low ends of the 10-point scale. This suggests that a dichotomous (yes/no) measure may be better for assessing these activities in a future trial. Finally, while digitally delivered interventions provide access to those who may have limited ability to travel, they do necessitate access to the internet which is an inherent limitation. No one screened for this study was excluded due to inability to access the Internet.

Despite these limitations, this study had several strengths. It had a high participant retention rate (96.6%), and it established the feasibility of a digitally delivered, integrated gardening, cooking, and nutrition education curriculum which provides a full-lifecycle, garden-to-plate perspective for engaging in healthy lifestyle behaviors. In addition, the systematic reporting of qualitative post-program participant recommendations represents a significant strength that will improve the future intervention. Areas of focus will include improving intervention activities for gardening activities that participants were less likely to achieve (spring garden, fall garden, soil test) and specific participant-identified content areas.

## Conclusion

A digitally delivered, multiple behavior change gardening intervention for adults with CVD risk factors was feasible, acceptable to participants, and they had meaningful changes in behavioral mediators. The next steps are to refine the intervention based on participant recommendations and test it in a future randomized controlled trial.

## Data Availability

The datasets used and/or analyzed during the current study are available from the corresponding author on reasonable request.
